# Injectable and photocurable macromonomers synthesized using a heterometallic magnesium–titanium metal–organic catalyst for elastomeric polymer networks†

**DOI:** 10.1039/d3ra02157b

**Published:** 2023-06-19

**Authors:** Malwina J. Niedźwiedź, Wojciech Ignaczak, Peter Sobolewski, Agata Goszczyńska, Gokhan Demirci, Miroslawa El Fray

**Affiliations:** a Department of Polymer and Biomaterials Science, Faculty of Chemical Technology and Engineering, West Pomeranian University of Technology in Szczecin Al. Piastów 45 70-311 Szczecin Poland mirfray@zut.edu.pl gdemirci@zut.edu.pl

## Abstract

Injectable and *in situ* photocurable biomaterials are receiving a lot of attention due to their ease of application *via* syringe or dedicated applicator and ability to be used in laparoscopic and robotic minimally invasive procedures. The aim of this work was to synthesize photocurable ester-urethane macromonomers using a heterometallic magnesium-titanium catalyst, magnesium-titanium(iv) butoxide for elastomeric polymer networks. The progress of the two-step synthesis of macromonomers was monitored using infrared spectroscopy. The chemical structure and molecular weight of the obtained macromonomers were characterized using nuclear magnetic resonance spectroscopy and gel permeation chromatography. The dynamic viscosity of the obtained macromonomers was evaluated by a rheometer. Next, the photocuring process was studied under both air and argon atmospheres. Both the thermal and dynamic mechanical thermal properties of the photocured soft and elastomeric networks were investigated. Finally, *in vitro* cytotoxicity screening of polymer networks based on ISO10993-5 revealed high cell viability (over 77%) regardless of curing atmosphere. Overall, our results indicate that this heterometallic magnesium-titanium butoxide catalyst can be an attractive alternative to commonly used homometallic catalysts for the synthesis of injectable and photocurable materials for medical applications.

## Abstract 2

Injectable and *in situ* photocurable biomaterials are receiving a lot of attention due to their ease of application *via* syringe or dedicated applicator and ability to be used in laparoscopic and robotic minimally invasive procedures.

## Graphical abstract

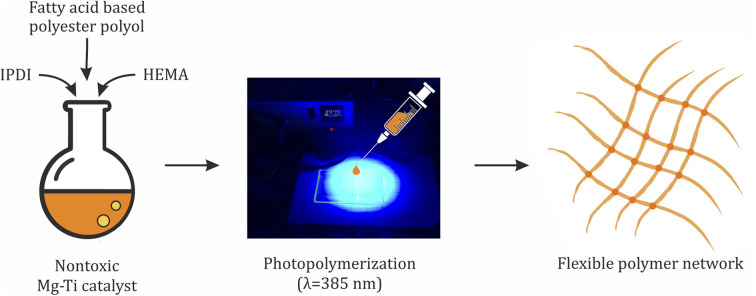

## 1 Introduction

Photocurable polyurethanes are gaining great interest for use in medical applications, particularly for minimally invasive surgical procedures. For example, these types of novel biomaterials can be in the form of injectable (macro)monomers that can be transformed by UV light into elastomeric patches.^1^ As a result, there is a research focus into new formulations and materials with improved properties, new synthesis routes, and new catalytic systems. The most commonly used catalysts for the synthesis of polyurethanes include antimony trioxide,^2^ antimony acetate^3^ and ethylene glycol aluminium.^4^ However, these homometallic catalysts are toxic and thus require difficult and costly post-processing to remove residual catalyst, particularly when the product is intended for medical applications.^5,6^ Tin-based catalysts are another class of popular and effective catalysts for the synthesis of polyurethanes.^7–9^ One commonly used example is dibutyltin dilaurate (DBTDL),^10^ which is also used for the synthesis of polyesters^11,12^ and many other elastomers, especially for coating applications.^13^ However, tin-based compounds are also toxic (with toxicity threshold of 3 ppm (ref. 14)), and thus they are also difficult and costly to remove from many polymers, including poly(urethane-amides),^14,15^ polyurethanes^13^ or polylactides.^16^ Therefore, there is a need to develop new and non-toxic—or at least less toxic—compounds for the synthesis of polymers and polymer networks, especially for food packaging or the medical industry.

In our previous work, we demonstrated that bismuth and zinc-based homometallic catalysts can be attractive, less toxic alternatives to DBTDL for the synthesis of photocurable ester-urethane macromonomers for elastomeric networks preparation. In that work, we discussed there two possible catalysis mechanisms: insertion and Lewis acid.^17^ Importantly, because isophorone diisocyanate (IPDI) is an asymmetric cycloaliphatic diisocyanate with primary and secondary isocyanate groups, the choice of catalyst plays an important role. Typically, the secondary cycloaliphatic group is more reactive than the primary aliphatic group.^18^ For the case of Lewis acid catalysts (such as DBTDL), the reactivity of the secondary isocyanate group is further increased. Meanwhile, for the case of Lewis base catalyst (such as DABCO), the primary isocyanate group becomes slightly more reactive, leading to similar reactivity of both NCO groups.

Recently there has been growing interest in heterometallic complexes which can operate *via* multiple interactions and can result in higher catalytic activity compared to their homometallic analogues.^19^ For example, heterometallic complexes of Ti/Zn,^20^ La/Mg,^21^ and Li/Mg^22^ have been shown to have superior catalytic activities to their homometallic analogues for the ring-opening polymerization. Most of these complexes feature a M–O–M′ framework, enabling intermetallic electronic communication and/or “ate”-type activation (*vide supra*), which can lead to enhanced nucleophilicity of the M–R bond (*e.g.* R = alkoxide). Thus far, experimental observations presented in the above, as well as many other works suggest that metals in multi-metallic catalytic systems have different functions: (1) larger and more electropositive metals (*e.g.* potassium, lithium, sodium) act as monomer coordination sites; (2) smaller and less electropositive metals (*e.g.* magnesium, zinc, aluminium, indium, yttrium, germanium, tin) act as the source of metal-alkoxide nucleophiles.^19^

**Fig. 1:**
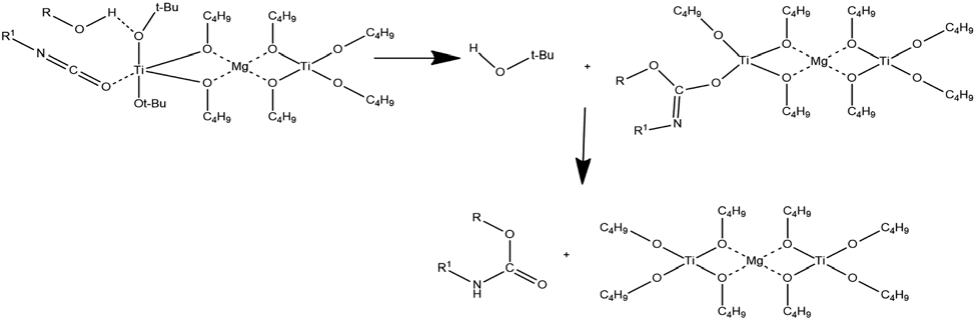
Scheme of magnesium-titanium butoxide catalyst mechanism.

In this context, a potentially promising heterometallic catalytic system is magnesium-titanium(iv) butoxide. This catalytic system has been already used for different type of reactions, including polycondensation^23^ and polyesterification^24–26^ of polyamides,^23,25^ poly(ester-ether)s,^27,28^ poly(butylene terephthalate) (PBT)-based,^23,29–31^ and poly(butylene-succinate) (PBS)-based copolymers.^32^ Unfortunately, the organo-bimetallic magnesium-titanium catalytic mechanism is not well described. However, it can be assumed that the isocyanate is activated by coordination to the Lewis acid, while the alcohol is activated through a hydrogen bond with a basic *tert*-butyl alcohol ligand. The alcohol is thus delivered through a six-membered transition state. The methalated carbamate then captures a proton from tertbutyl alcohol or from the added alcohol (Fig. 1).^33^

Building upon our previous work, here we investigated synthesis of the telechelic macromonomers based on polyester diol using magnesium-titanium(iv) butoxide as catalyst. We hypothesized that this heterometallic catalytic system would enhance selectivity toward the secondary isocyanate function of IPDI and would result in shorter reaction times than our previous work with bismuth and zinc-based homometallic catalysts,^17^ while at the same time obtaining one dominant macromonomer that would remain non-toxic. Towards this aim, we examined chemical structure and physio-chemical properties of the obtained macromonomers and we performed the cytotoxicity screening of fabricated elastomeric networks to assess their suitability for medical applications, especially for delivery of a precursor in minimally invasive medical procedures.

## 2 Experimental

### 2.1. Material and reagents

Priplast 1838 (dimer linoleic acid based polyester diol) was kindly provided by Cargill Bioindustrial (The Netherlands). Isophorone diisocyanate (98%, mixture of isomers) (IPDI) and 2-hydroxyethylmethacrylate (97%) (HEMA) were purchased from Merck KGaA (Germany). Phenothiazine (PTZ) was purchased from Sigma Aldrich (Poland). Photoinitiator Omnirad 819 was purchased from IGM resins (The Netherlands). Ethyl acetate (EtOAc) and methanol were purchased from Chempur (Poland). HEMA was used after distillation under reduced pressure; all other reagents were used as received. Magnesium chips and titanium(iv) butoxide were purchased from Sigma Aldrich (Poland), and *n*-butanol was purchased from Eurochem BGD (Poland). Murine fibroblast cell line, L929, as well as all cell culture reagents were purchased from Sigma Aldrich (Poland). All disposable cell culture plasticware was purchased from VWR (Poland).

### 2.2. Synthesis of magnesium-titanium(iv) butoxide catalyst

In order to synthesize the magnesium-titanium(iv) butoxide catalyst, 0.14 g of magnesium chips were added to 30 mL of *n*-butanol in a 100 mL round-bottom flask and heated to 117 °C for 4–6 h. Then, 3.60 g of titanium(iv) butoxide was added to the flask and the set temperature was maintained for an additional 1 h. After this, the flask was purged with argon and left overnight. Then, the mixture was centrifuged and supernatant was collected. The chemical structure of the obtained catalyst was characterized using NMR (ESI Fig. S1†).

### 2.3. Synthesis of telechelic macromonomers containing fatty acid moieties

**Fig. 2:**
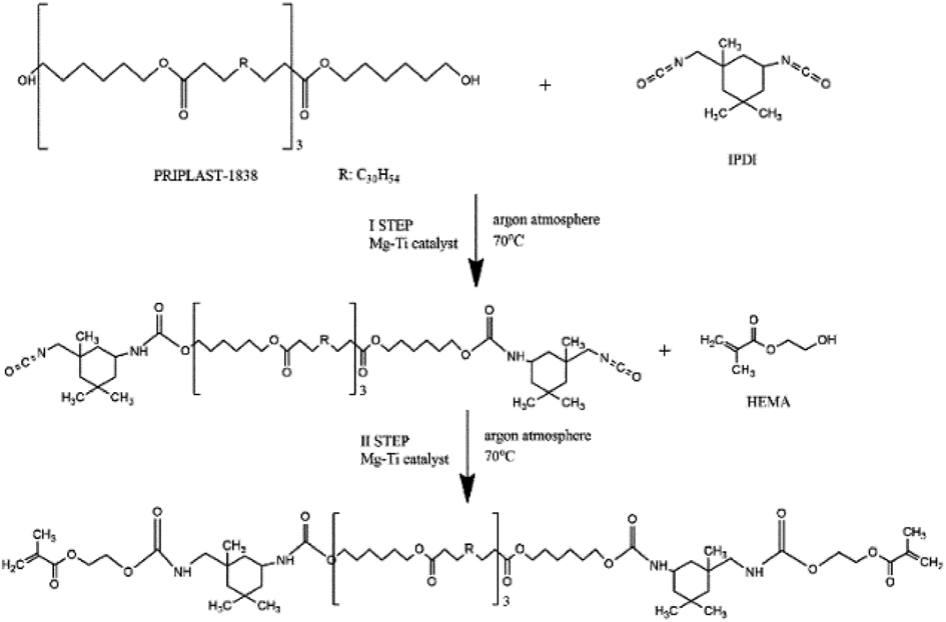
Scheme of the synthesis of telechelic macromonomer containing ester and urethane groups.

Synthesis of telechelic macromonomers was performed in two steps based on our previous work^17^ and the reaction scheme is presented in Fig. 2. In the first step, 50 mL of EtOAc in a 250 mL round-bottom flask was degassed with three argon/vacuum cycles. Then, the flask was placed in an ice bath and different amounts of catalyst (0.1, 0.25, 0.50, and 1 mol% calculated with respect to Priplast OH groups) and 13 mL of IPDI were added. After that, 50 g of Priplast (P1838), dissolved in 50 mL of EtOAc, was added dropwise, the flask was immersed into an oil bath, and the reaction was carried out at 70 °C in argon atmosphere. The second step was started when the ratio of *A*_2262_/*A*_1526_ of decaying N=C=O groups and forming urethane bonds (bending N–H vibrations), as calculated from FT-IR spectra, reached approx. 5. Based on NCO titration, this value corresponds to approx. 50% of reacted groups. For the case of the synthesis with use of the 0.1 mol% of catalyst, the ratio stabilized at approx. 10, therefore the second step was started with this higher ratio. Then, 6 mg of PTZ (inhibitor), a second dose of catalyst (the same amount as in the first step), and 13.2 mL of HEMA were added to the reaction mixture. The reaction was considered finished when all of the isocyanate groups were converted, as determined using FT-IR (absence of the band at 2262 cm^−1^). The flask was then removed from the oil bath and allowed to cool down to room temperature. The product was purified from unreacted reagents by triple precipitation in four-fold excess of ice-cold methanol and any residual solvents were evaporated under reduced pressure.

### 2.4. Photopolymerization towards elastomeric networks

Photopolymerization of the obtained macromonomers was performed according to the procedure thoroughly described in our previous work.^17^ Briefly, the obtained telechelic macromonomers were mixed with a 2% w/w solution of Omnirad 819 photoinitiator in EtOAc. Then, the solvent was evaporated under reduced pressure and the obtained highly viscous liquid was poured onto a glass plate and was spread into a film with a steel applicator. In this manner, elastomeric films 1 mm of thickness were obtained. Next, the elastomeric networks were irradiated with a UVA light source (*λ*_peak_ = 385 nm; DYMAX Bluewave LED Prime UVA) for 150 s at a maximum intensity 20 mW cm^−2^. The process was carried out in a glove box under air and argon atmosphere.

### 2.5. Methods

The chemical structure of the obtained materials and progress of the reactions were monitored by using Fourier transform infrared spectroscopy (FTIR) using a BRUKER ALPHA Platinum spectrometer (Germany), at room temperature (4000–600 cm^−1^, resolution 2 cm^−1^, 32 scans). Liquid samples were analysed in transmission mode, after pouring the samples between sodium chloride plates. Spectra of the cured films were collected using attenuated total reflection (ATR) cell with diamond crystal. EZ OMNIC software was used for data analysis.

Nuclear magnetic resonance spectroscopy (^1^H and ^13^C NMR) was performed to confirm the chemical structure of the obtained telechelic macromonomers. Spectra were recorded using Bruker DPX HD-400 MHz at 25 °C. CDCl_3_ was used as solvent and all shifts were determined with respect to tetramethylsilane (TMS). Additionally, the degree of acrylation^34^ was calculated based on proton NMR according to eqn (1):

1 Degree of acrylation = δ_u,w_ × (4/δ_a_)

where δ_u,w_ represents the average signal resulting from the acrylate protons, and δ_a_ the signal resulting from protons a in the Priplast 1838 (see Fig. 4 for peak assignments).

Dynamic viscosity of the macromonomers was assessed using a DV3TRV rotary cone-plate rheometer (Brookfield AMETEK, USA). The parameters for the measurement were as follows: measuring head in the cone-plate system with a diameter of *ϕ* = 40 mm, distance between cone and plate *h* = 1 mm, deformation of 30%, constant shear rate *γ̇* (s^−1^) 0.200, and temperature 25 °C.

Differential scanning calorimetry analysis (DSC) was performed using Q2500 DSC (TA Instruments, New Castle, Delaware, USA) calorimeter to examine phase change behaviour of macromonomers. Samples were weighed (∼5 mg) into aluminium pans and hermetically sealed before the analysis. Samples were cooled down to −90 °C, held isothermally for 3 min, and then heated up to 120 °C. The heating/cooling rate was 5 °C min^−1^. The data were analysed using TRIOS software.

Gel permeation chromatography (GPC) was used to determine average molar (apparent) mass (*M̄*_n_ and *M̄*_w_) and molar mass dispersity, *Ð* (*M̄*_w_/*M̄*_n_) of obtained macromonomers. The GPC Wyatt (Germany) instrument was composed of a guard column and four Perfect Separation Solutions (PSS) columns (50, 100, 1000, and 100 000 Å), Dn 2010 WGE Dr Bures differential refractometer (RI), and Wyatt MALLS DAWN HELEOS multi-angle light scattering (LS) detectors. 8 different linear polystyrene standards (molecular weight of 970, 1990, 5030, 10 680, 19 760, 34 800, 70 950, and 126 700 g mol^−1^ (Polymer Laboratories and Solutions LLC, USA)) were used for calibration of the equipment. The tetrahydrofuran was used as the mobile phase at a flow rate of 1 mL min^−1^ at 35 °C. PSS WinGPC Unity software was used for data collection and molecular mass calculations.

In order to assess gel fraction of photocured elastomeric networks, samples were refluxed for 6 hours in a Soxhlet extraction apparatus (Behr Labor-Technik, Germany) in EtOAc. The samples were weighed before extraction (*W*_initial_) and after drying (under reduced pressure until a constant mass was achieved) (*W*_final_). The gel fraction was calculated according to eqn (2):

2 Gel fraction (%) = *W*_final_/*W*_initial_ × 100

Swelling tests of photocured materials were performed according to EN ISO:175. Discs (1.6 cm in diameter) were placed in chlorobenzene for 24 h at 40 °C. After that time, samples in closed laboratory glass vessels were removed from the incubator and left for 20 min at room temperature to cool down (15–30 min). The discs were then washed with THF, dried with filter paper, and weighed. The weighing after removal from the solvent took no longer than 30 s. The swelling ratio was calculated according to eqn (3), where *m*_s_ indicates for swollen mass and *m*_d_ is dry mass of the sample.

3 Swelling (%) = (*m*_s_ − *m*_d_)/*m*_d_ × 100

Dynamic mechanical thermal analysis (DMTA) measurements of photocrosslinked films were performed using a Q800 DMTA instrument (TA Instruments). Samples were measured at a frequency of 1 Hz and amplitude of 1 μm. Samples were first cooled to −90 °C and then heated to 120 °C. The rate of heating was set to 3 °C per minute.

Finally, elastomeric polymer networks were screened for cytotoxicity according to ISO10993-5 (ref. 35) using murine fibroblast cell line L929, as described previously.^17^ Extracts were prepared in duplicate, by incubating samples of each photocured material (3 cm^2^ area, thickness 0.5 mm, cut into small pieces) in 1 mL of complete growth medium (Dulbecco's Modified Eagle Medium (DMEM), containing 10% Fetal Bovine Serum (FBS), 2 mM l-glutamine, 100 U mL^−1^ penicillin, and 100 μg mL^−1^ streptomycin) for 24 hours at 37 °C, in a 24-well plate. At the same time, 10 × 103 L929 cells (passages 10–25) were plated per well in a 96-well plate in complete growth medium and incubated for 24 hours in a cell culture incubator. After 24 hours, the medium was aspirated and replaced with 100 μL of extract, followed by an additional 24 hours of incubation (5 technical replicates). Cell viability was then assessed using light microscopy (Delta Optical IB-100, Mińsk Mazowiecki, Poland) and the resazurin viability assay.^36^ A multi-functional plate reader (Biotek Synergy HTX, Winooski, VT, USA) was used to measure the fluorescence signal (excitation 540 nm, emission 590 nm). The experiments were repeated with similar results. The cell viability was expressed as percent of sham-extract treated cells.

## 3 Results and discussion

### 3.1. Progress of the reaction of macromonomers synthesis

Progress of the reactions was monitored using FTIR, as described in our previous study.^17^ Briefly, the first step of the reaction was considered to be completed when the NCO/NH ratio reached approx. 5, which corresponds to ∼50% of NCO groups being consumed and ensuring the complete conversion of –OH groups. In comparison to our previous work, where the range was approx. 3–6, here the range of values for the (*A*_2262_)/(*A*_1526_) ratio was similar with exception of 0.1 mol% catalyst concentration.

**Fig. 3:**
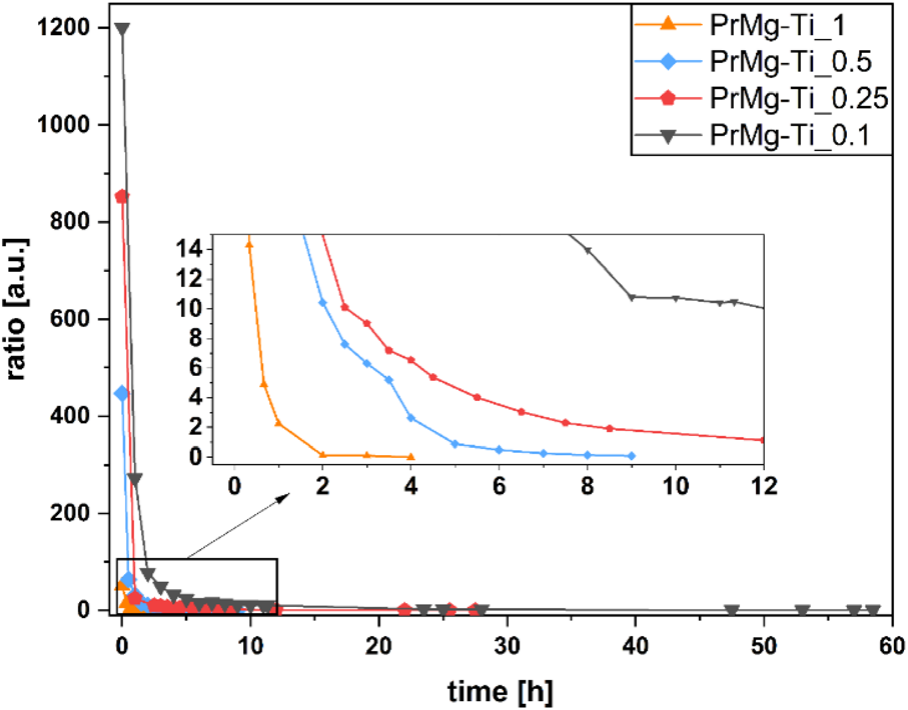
Kinetics of macromonomers synthesis performed with use of different concentrations of Mg–Ti butoxide catalyst.

Therefore, the observed differences in kinetics relative to our previous work may be explained by differences in selectivity and activity of a catalyst. Further, the higher ratio (∼11) NCO/NH observed for the case of synthesis using 0.1 mol% of catalyst may be also related to the pot life of Mg–Ti butoxide catalyst: at this low concentration the reaction proceeds slowly and the catalytic activity of the catalyst may begin to decrease before the lower ratio can be reached. All of the FT-IR spectra for all reactions are available in the ESI, Fig. S2–S5.† The kinetics of all reactions, shown as ratio of absorbance bands (*A*_2262_) to (*A*_1526_) as a function of time, are presented in Fig. 3. Meanwhile, Table 1 presents the reaction times and yields.

**Table 1:** Summary of the reaction parameters

Material	Catalyst concentration (per step) [mol%]	Reaction time (I step) [hours]	*A*_2262_/*A*_1525_ ratio after I step	Reaction time (II step) [hours]	Total reaction time [hours]	Reaction yield [%]
PrMg-Ti_0.1	0.1	11	10.43	47.5	58.5	53
PrMg-Ti_0.25	0.25	4.5	6.53	23.5	28	67
PrMg-Ti_0.5	0.5	3.5	5.21	5.5	9	57
PrMg-Ti_1	1	0.7	4.91	3.3	4	63

As it can be seen from Fig. 3 and Table 1, as can be expected, higher concentrations of catalyst resulted in shorter total reaction time (58.5 h for 0.1 mol% and 4 h for 1 mol%) and lower ratio of (*A*_2262_)/(*A*_1525_) after the first step (10.43 for 0.1 mol% and 4.91 for 1 mol%). The reaction yields are similar, ranging from 53% to 67%, which is consistent with our previous work where we observed a similar range (62–70%).^17^

Importantly, compared to our previous work,^17^ here the total reaction time for the highest concentration of Mg–Ti butoxide catalyst was markedly shorter, as compared to homometallic bismuth and zinc catalysts (7 hours and 25 hours, respectively)—despite a lower catalyst concentration (1 mol% *vs.* 2 mol%). This confirms that a different catalytic mechanism was involved for In the previous work,^17^ the homometallic catalysts were acting *via* either insertion mechanism or as Lewis acids.^17,38^ Meanwhile, in the heterometallic system used here, we hypothesize that the catalyst acts similarly to titanium alkoxide catalyst *via* insertion of the isocyanate into the Ti–O bond of the alkoxy ligand, but that due to the higher stability of the heterometallic catalyst, the pot life of the catalyst is elongated.^33,37,38^

### 3.2. Chemical structure of macromonomers by IR and NMR spectroscopy

**Fig. 4:**
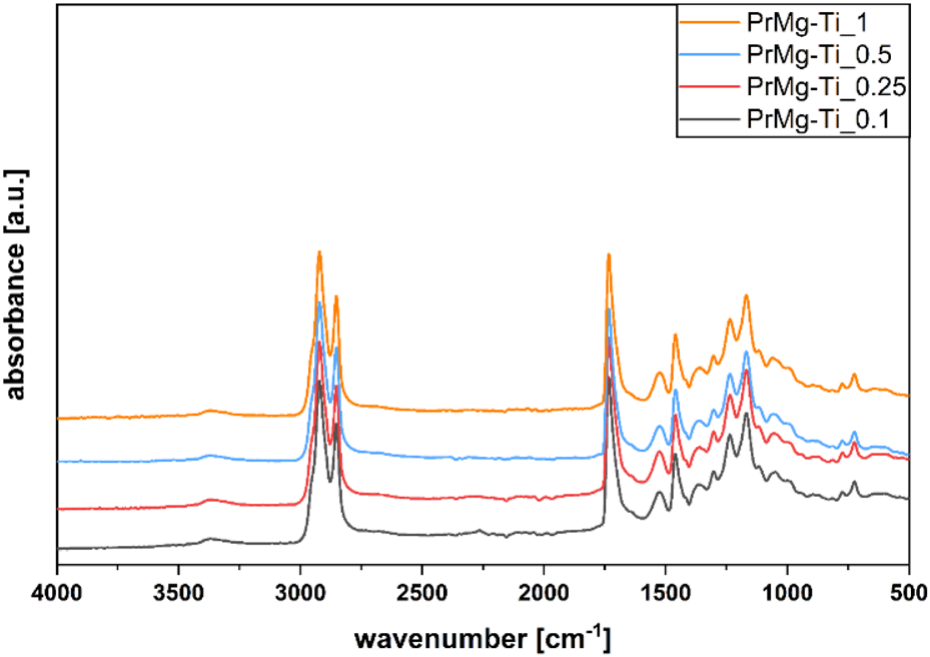
FT-IR spectra of macromonomers obtained with use of various concentrations of Mg–Ti catalyst.

In order to confirm the chemical structure of the obtained macromonomers we used FT-IR spectroscopy. All of the obtained spectra were similar, indicating that the obtained macromonomers had the same chemical structure, in line with expectations, featuring the presence of both ester and urethane groups with double bond termini (Fig. 4).

The presence of absorbance bands at 3368 and 1525 cm^−1^ confirmed IPDI incorporation (corresponding to stretching vibrations of N–H in urethane groups). The band at 1643 cm^−1^ corresponding to stretching vibration of C=C, confirmed the presence of HEMA. Additionally, the bands at 2921 and 2853 cm^−1^ correspond to stretching vibrations of C–H in –CH_2_ and –CH_3_. The band 1732 cm^−1^ is characteristic for stretching of C=O in ester bonds of the polyester diol, urethane and methacrylic groups. The band at 1460 cm^−1^ is characteristic for R–CH_2_–CH_3_ and –C–C– in rings, while the band at 1369 cm^−1^ reflects rocking vibrations of C–H in fatty acid chains of Priplast 1838. At 1302 and 1049 cm^−1^, the presence of RCOOR′ vibration in esters was detected, while the bands at 1236 cm^−1^ and 1168 cm^−1^ reflect the –C–O– stretching vibration and –C–O–C(=O) stretching vibration, respectively.

^1^H NMR and ^13^C NMR spectroscopy were used as more in-depth methods of verifying the chemical structure of synthesized macromonomers. Overall, the analysis of NMR spectra confirmed the FT-IR findings. All of the obtained macromonomers had similar structure, consistent with our expectations based on prior work.^17^

**Fig. 5:**
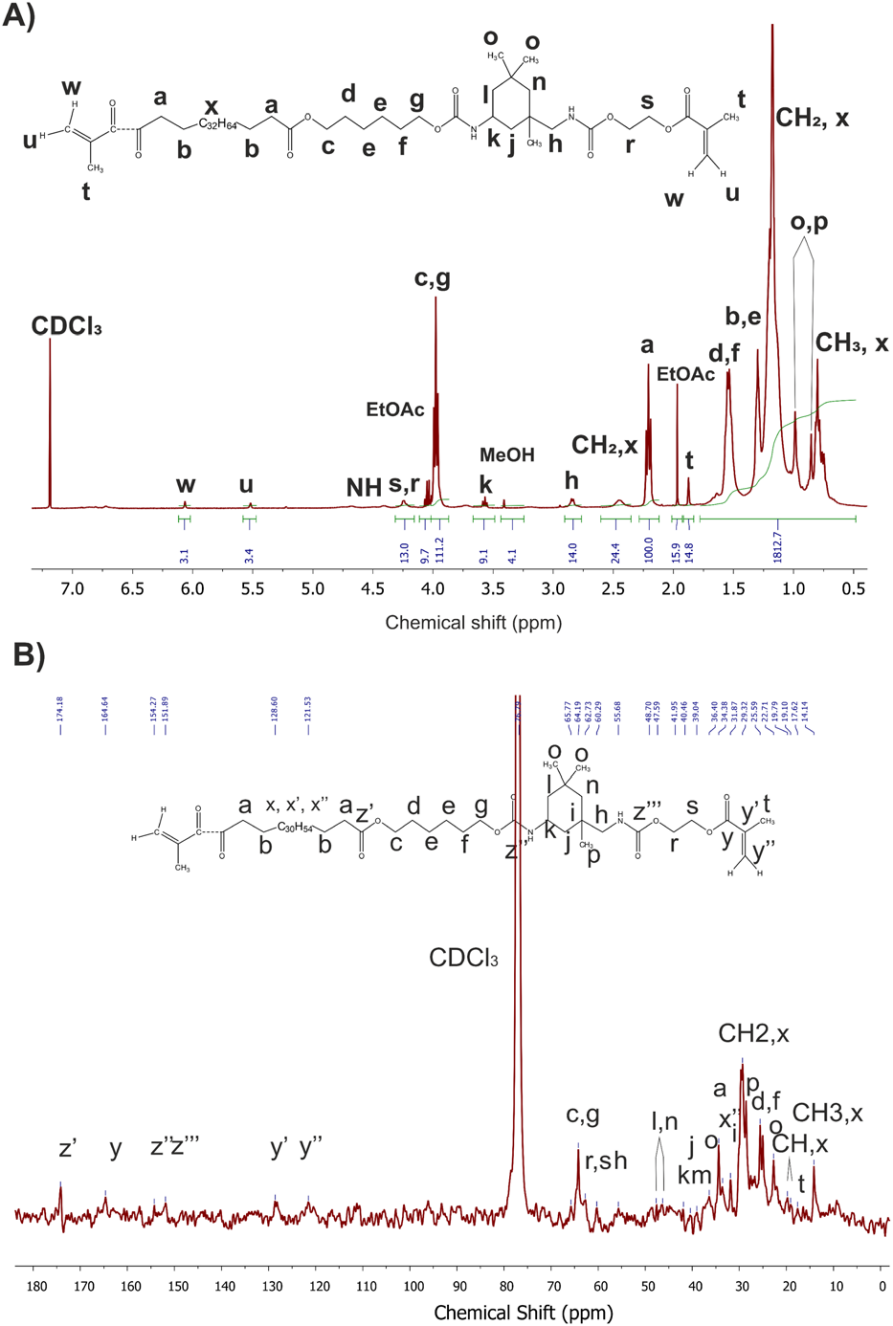
^1^H (A) and ^13^C NMR (B) spectra of macromonomers synthesized with of 0.1 mol% Mg–Ti butoxide catalyst.

As a representative example, Fig. 5 presents ^1^H and ^13^C NMR spectra of macromonomer obtained using 0.1 mol% Mg–Ti butoxide catalyst per step with peak assignments. The ^1^H NMR spectra for the other catalyst concentrations are available in the ESI (see Fig. S6†).

**Fig. 6:**
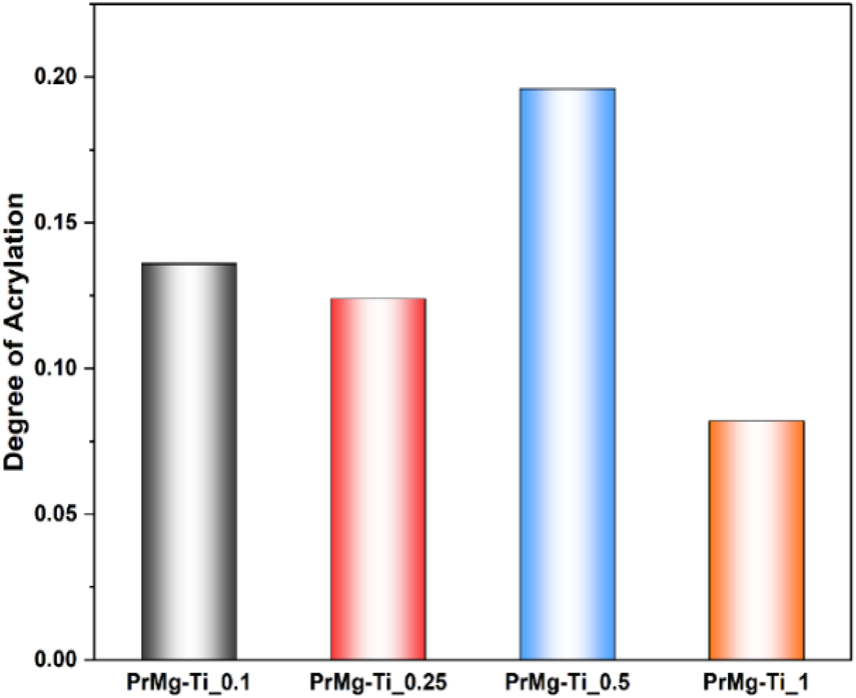
Degree of acrylation of macromonomers.

The degree of acrylation (Fig. 6 and Table S1†) was calculated based on NMR spectra which influences the crosslinking density of the obtained elastomeric networks. It can be observed that increasing the concentration of catalyst led to a decrease in the degree of acrylation, with the exception of sample PrMg-Ti_0.5 (0.5 mol% of catalyst). Higher catalyst concentration increases the reaction rate to achieve the equilibrium faster to the product which results in lower number of accessible NCO groups for methacrylate (HEMA) attachment. Lower catalyst concentration may result in reduced IPDI addition to polyester diol due to which some free terminal OH groups are still in the molecule which also leads to lower HEMA attachment. These data are also correlated with gel fraction of films after photocuring, which will be discussed in Section 3.7.

Unfortunately, obtained spectra did not allow us to explicitly support the presence one predominant macromonomer. We expected to note a shift of carbons and protons corresponding to IPDI, that would confirm that the Mg–Ti butoxide catalyst first increased the reactivity of the secondary isocyanate group.

### 3.3. Molecular mass and viscosity of macromonomers

The results of GPC analysis are presented in Table 2 and Fig. S7 in the ESI.† For all of the macromonomers obtained with different catalyst concentration, an increase of molecular mass and decrease of dispersion was observed, as compared to the starting polyester diol, Priplast 1838. The results presented in Table 2 clearly show that *M̄*_n_ and *M̄*_w_ values were lower with increasing catalyst concentration. The higher catalyst concentrations shorten the total reaction time substantially, because the reaction proceeds simultaneously at a greater number of active centers of the catalyst. At the same time, this effect favours the formation of shorter macrochains. Overall, the dispersity indexes of the macromonomers are low (1.5–1.7), which is consistent with our hypothesis that the Mg–Ti butoxide catalyst would favour formation of one predominant macromonomer.

**Table 2:** GPC results and dynamic viscosity for the synthesized macromonomers

Material	Catalyst concentration (mol%)	*M*_n_ [g mol^−1^]	*M*_w_ [g mol^−1^]	*Ð* [*M*_w_/*M*_n_]	Dynamic viscosity [Pa s]
Priplast 1838	—	3700	7800	2.12	18 ± 2
PrMg-Ti_0.1	0.1	10 000	17 500	1.74	172 ± 12
PrMg-Ti_0.25	0.25	8200	14 100	1.71	162 ± 28
PrMg-Ti_0.5	0.5	8200	13 000	1.58	136 ± 15
PrMg-Ti_1	1	7500	11 400	1.53	119 ± 38

**Fig. 7:**
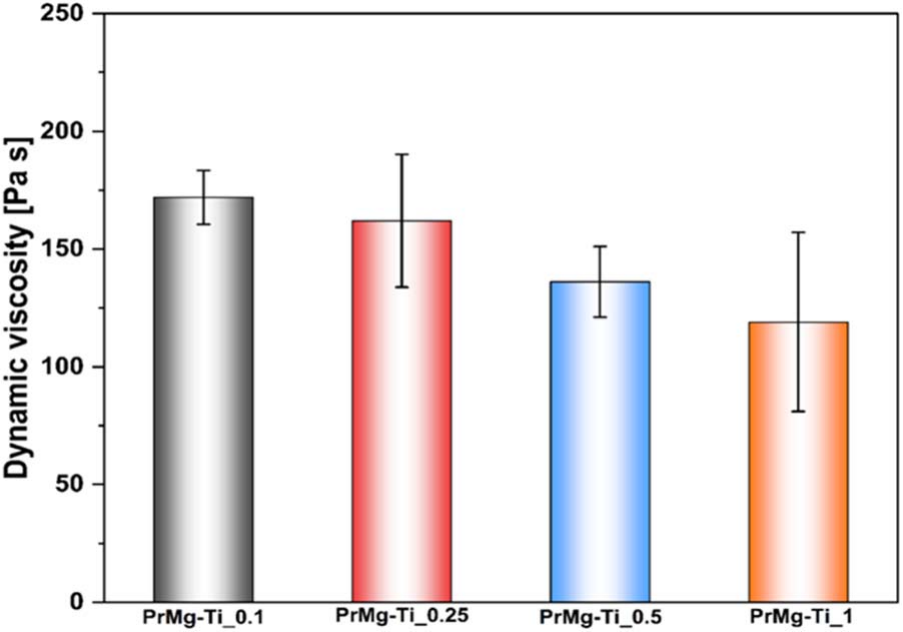
Dynamic viscosity for macromonomers synthesized with different concentrations of Mg–Ti butoxide catalyst.

As expected, the dynamic viscosity of the macromonomers reflects the same trend (Fig. 7 and Table 2), with a decrease from 172 Pa s for the material with the lowest concentration of the catalyst to 119 Pa s for material synthesized with the highest catalyst concentration.

### 3.4. DSC of macromonomers

**Fig. 8:**
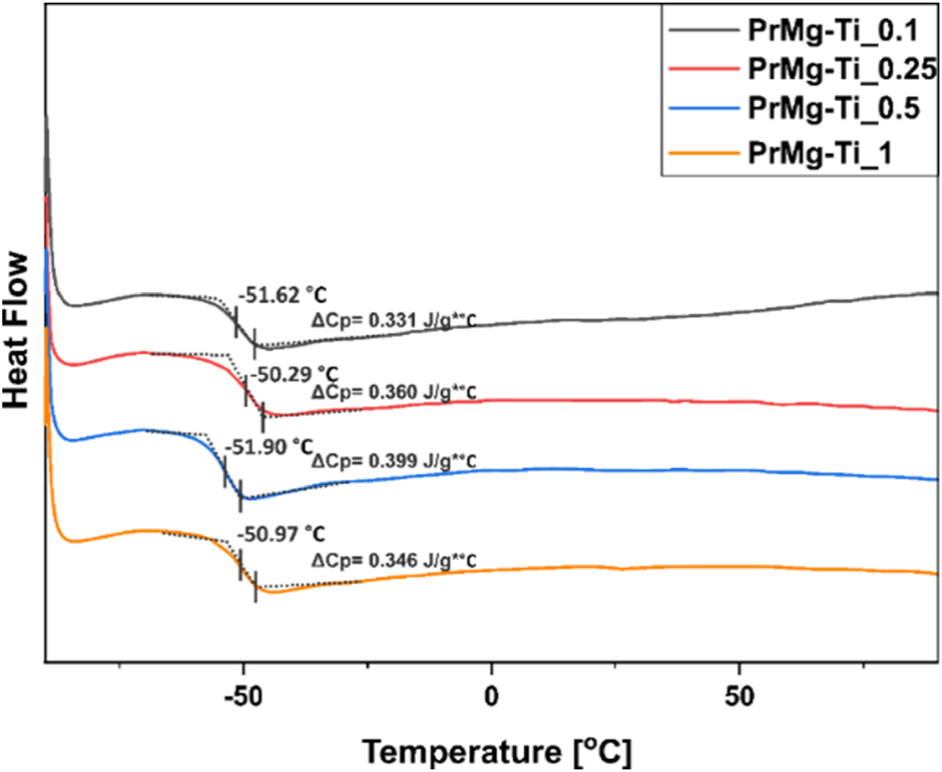
DSC thermograms of macromonomers synthesized with different concentrations of Mg–Ti butoxide catalyst.

DSC was used to determine the phase transitions of the obtained macromonomers. According to the results, the changes in catalyst concentration did not affect the main phase transition of the obtained macromonomers. As presented in Fig. 8, the glass transition temperatures (*T*_g_) and changes in heat capacity (Δ*C*_p_) for all materials were very similar: approximately −51 °C and 0.37 J g^−1^ °C (ESI Table S2†), respectively.

### 3.5. Chemical structure of photocrosslinked elastomeric networks

**Fig. 9:**
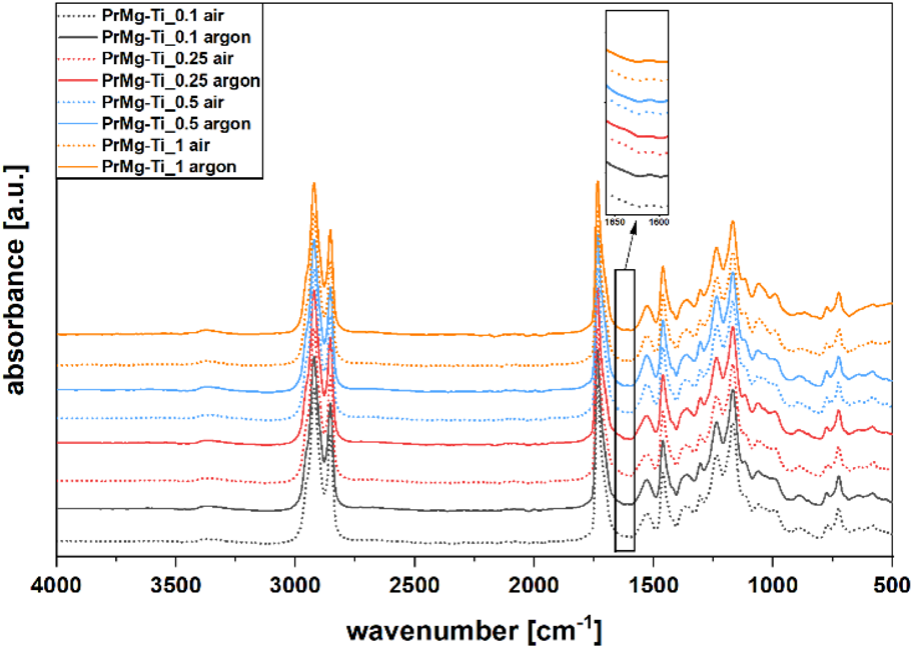
FT-IR spectra of cured films.

In order to confirm the functionality of the synthesized macromonomers, all of the obtained macromonomers were photocrosslinked under both air and argon atmospheres. The same amount (2% w/w) of the photoinitiator (Omnirad 819) was added for all of the obtained macromonomers and the photocuring process parameters were all the same. The chemical structure of elastomeric photocured films was verified using ATR-FTIR spectroscopy. Importantly, the spectra of all of the photocured elastomeric materials were similar, regardless of curing atmosphere (Fig. 9).

In all of the spectra presented in Fig. 9, similar bands related to the relevant groups were present. The decrease in the band intensity at 1643 cm^−1^, corresponding to the stretching vibration of C=C in methacrylate group, is evidence for the reaction of the unsaturated functional groups during the photopolymerization. Additionally, due to the reaction of methacrylate functional groups, a new band appears at 1634 cm^−1^, corresponding to the resultant C–C bond. For the case of materials that were cured under air atmosphere, we note that the signal at 1643 cm^−1^ is more pronounced, which is likely due to oxygen inhibition.

### 3.6. Gel fraction and swelling test of the photocrosslinked elastomeric networks

**Fig. 10:**
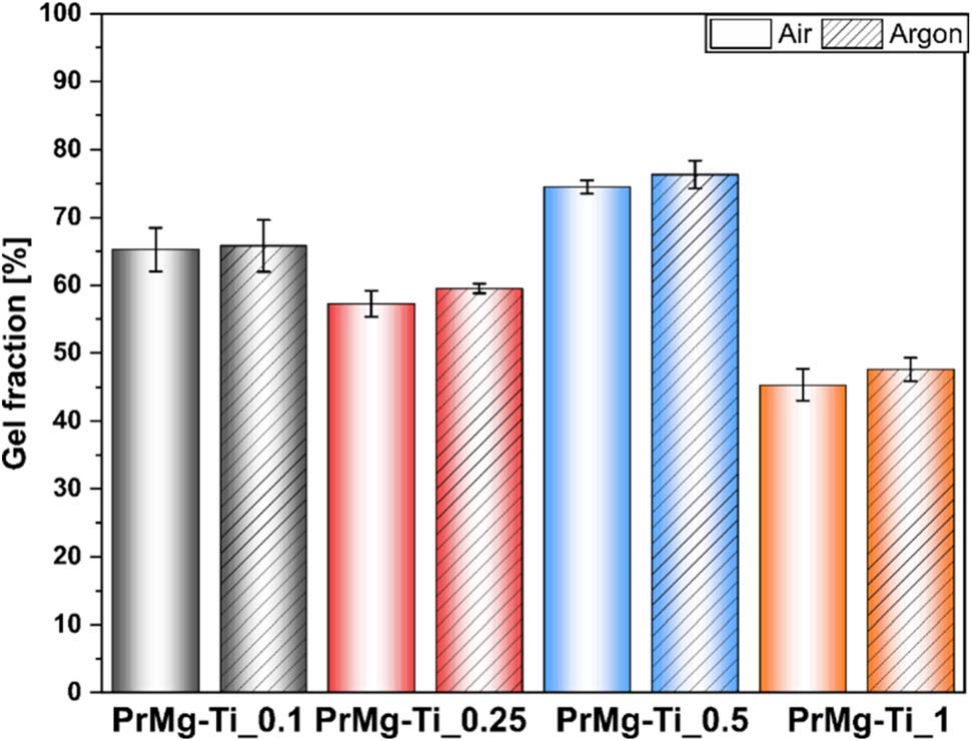
Gel fraction results of photocured thin films.

After photocuring under both atmospheres, the obtained elastomeric films showed relatively low values of gel fraction (see Fig. 10 and Table S3†), as compared to our previous results with homometallic Zn and Bi catalysts, where the gel fraction was ∼93%.^17^ Importantly, as in previous paper only a slight difference in gel fraction was observed between the two curing atmospheres. The highest value obtained for PrMg-Ti_0.5 (75%) and the overall trend was similar to that for the results of the degree of acrylation. The higher degree of acrylation for the macromonomers synthesized with this catalyst concentration (0.5 mol%), was responsible for a greater degree of crosslinking. As a result, due to diffusion limitations, those materials were less prone to solvent permeation and had a higher gel fraction. Likewise, the lowest gel fraction was observed for polymer network obtained from macromonomers synthesized with 1 mol% of catalyst, consistent with previously noted low acrylation degree. Ultimately, the higher the content of double bonds (acrylic groups) in the macromonomer structure, the denser polymer network. However, macromonomers with higher molar mass may also yield networks with higher crosslinking density, because the formation of free radicals takes place at ends of the growing chains. Longer chains may simplify the segmental diffusion of macromonomer ends, thus increasing the chance that an individual functional groups can react.

**Fig. 11:**
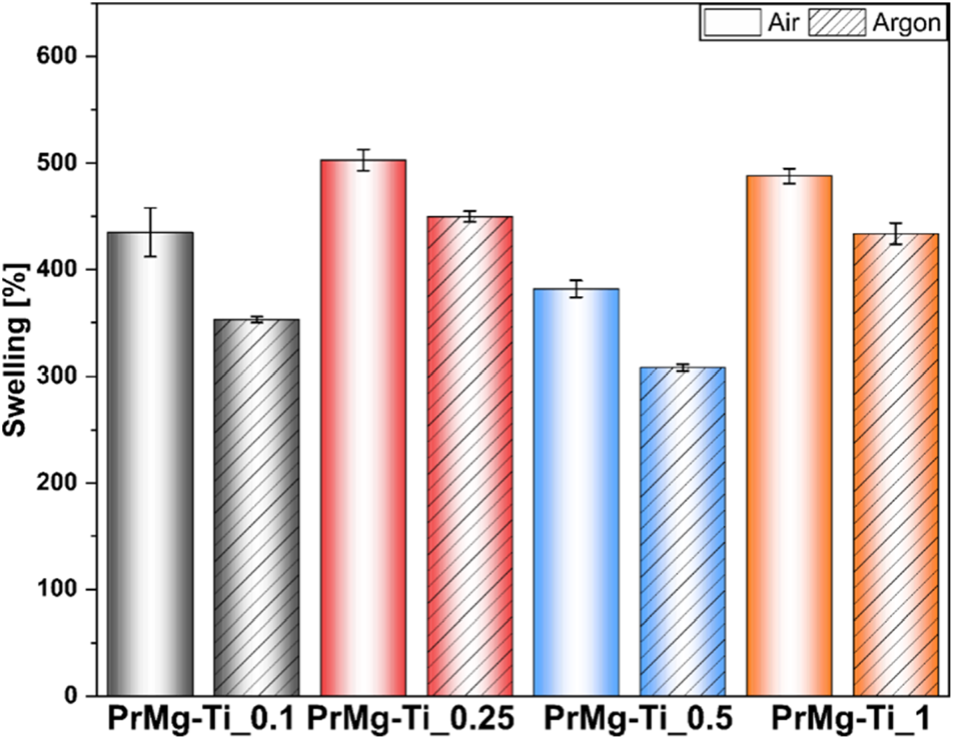
Swelling test results of obtained thin films.

To further assess the obtained photocured polymer networks, we performed swelling tests according to EN ISO:175 using chlorobenzene. The obtained results confirmed our hypothesis regarding the differences in the density of polymer networks. Further, in this case, the results (Fig. 11 and ESI Table S4†) clearly showed differences between the films cured under air and under argon, indicating an influence of oxygen inhibition on the curing process. The differences between the results arise from differences between the methods. In assessing gel fraction, the loss of material in solvent was measured, whereas in the swelling test the solvent uptake was determined.

### 3.7. DMTA

**Fig. 12:**
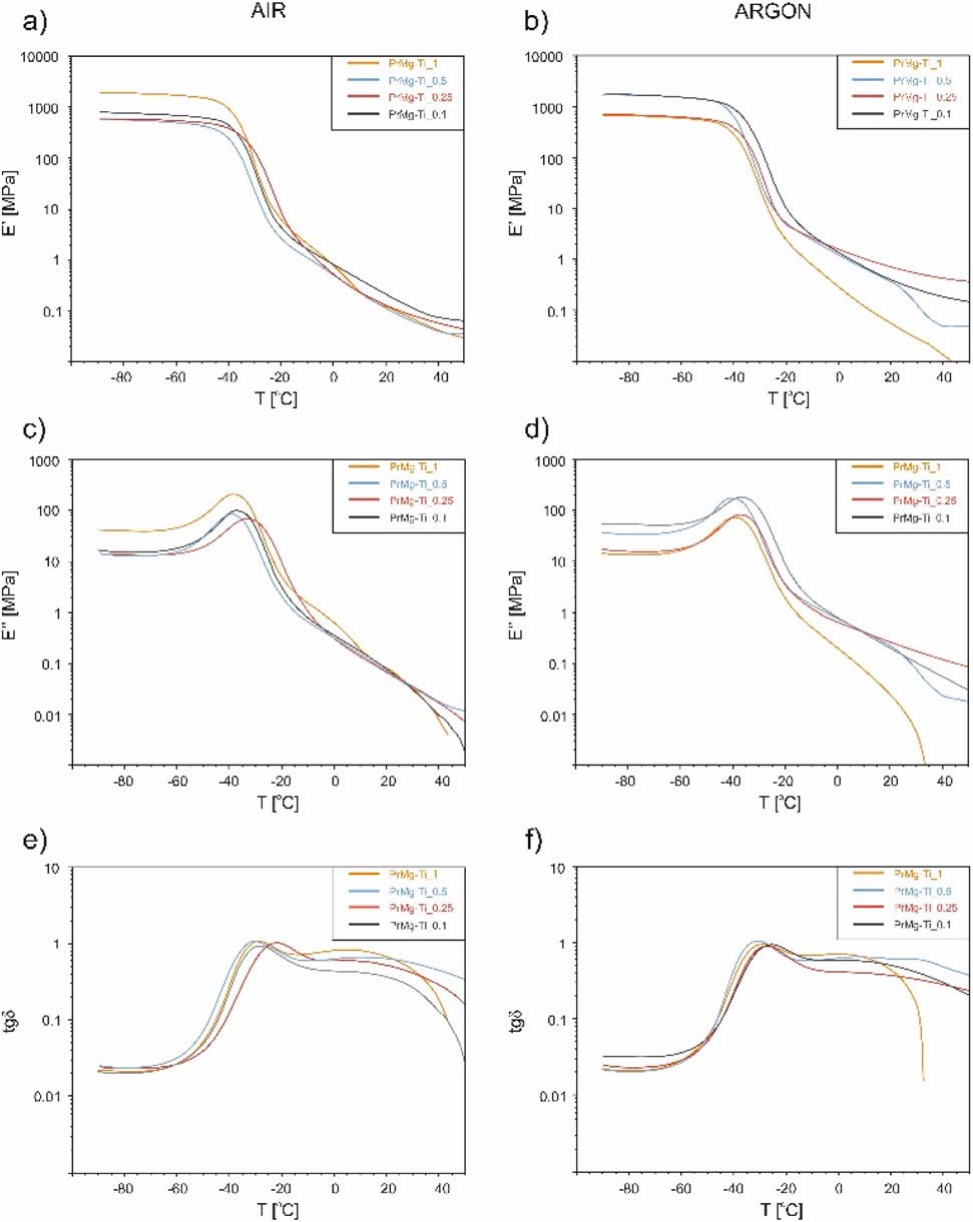
DMTA analysis of photocured polymer networks: (a and b) the storage modulus (*E′*), (c and d) loss modulus (*E′′*), and (e and f) the tangent of the phase angle (tan *δ*) of materials cured in air (a, c and e) and in argon (b, d and f).

Dynamic thermomechanical analysis (DMTA) was used to evaluate the relaxation changes in the amorphous phase of the photocured polymer networks obtained from the macromonomers synthesized with different concentration of the Mg–Ti butoxide catalyst (from 0.1 to 1 mol%). Additionally, we wanted to compare their mechanical profiles at 37 °C, from the point of view of potential medical applications. After photocuring using the same photoinitiator concentration and irradiation exposure, but under different atmospheres (air *vs.* argon), the obtained elastomeric thin films were subjected to dynamic mechanical thermal analysis in a tensile mode. The results, in a form of temperature dependence of the storage modulus (*E*′), loss modulus (*E*′′), and the tangent of the phase angle (tan *δ*) are presented in Fig. 12 (the numerical values are presented in Table S5†). Interestingly, the values of the storage modulus, *E*′ (Fig. 12a), representing elastic behavior in viscoelastic polymers, indicated that within the glassy state region (below −20 °C), there are some significant differences in polymers stiffness (values of *E*′ differ from ∼600 MPa to ∼1100 MPa), independent of the curing atmosphere and the catalyst concentration. After the phase transition, all of the polymer networks photocured under the same atmosphere exhibit comparable elasticity (more details in Table S5†). However, overall samples cured under argon were less elastic, as compared to those cured under air atmosphere (*i.e.* 0.2 *versus* 0.078 MPa for 0.1 mol% catalyst concentration). This effect is most likely due to the lower conversion of macromonomers for the case of samples photocured in air atmosphere, due the negative effect of oxygen inhibition. The resulting lower crosslinking density would result in more space for macrochains to arrange. Additionally, possible residual unreacted macroradicals, captured in the polymer network, can act as lubricants. Combined these two factors are likely responsible for the observed increase in elasticity. For the case of the sample prepared from macromonomers synthesized with 1 mol% of catalyst and photocured under argon atmosphere, fracture occurred during the measurement. This is likely due to its relatively low crosslinking density, related to a low degree of macromonomer acrylation. At high catalyst concentration (1 mol%), the isocyanate groups reacted with the hydroxyl termini very rapidly (stage I lasted less than 1 hour), facilitating the formation of shorter macrochains, which was confirmed by significant changes in the molecular weight and dynamic viscosity of this macromonomer. Additionally, at the same molar ratio of P1838 and IPDI maintained within the 1st step of the reaction, the obtained shorter chains will statistically have less NCO end-groups. This was likely to significantly influence the 2nd step of the reaction, resulting in lowest values of macromonomer acrylation degree.

Overall, the storage modulus values indicate (0.02–0.45 MPa) that the obtained elastomeric polymer networks can be suitable for soft tissue applications (the numerical values are presented in Table S5†). For reference, the values of storage modulus of human abdominal wall is in the range of 0.1–10 MPa, while the stiffness of the heart muscle at the end of diastole is between 0.2–0.3 MPa.^39^

In terms of the loss modulus (*E*′′) (a measure of viscous behavior in polymers), the results (Fig. 12b), showed only one relaxation maximum at ∼35 °C, corresponding to the amorphous phase of the polymer networks. Differences in relaxation temperatures were subtle (up to 5 °C), but a shift towards lower temperatures with increasing concentration of the catalyst used during the synthesis was noticed for both photocrosslinking atmospheres.

The changes in tan *δ*, a dimensionless parameter that does not represent any physical quantity directly, but is a measure the ratio of energy dissipated to energy stored with periodically variable deformation, are presented in Fig. 12c. The analysis of damping properties (relation of *E*′′ to *E*′) revealed that samples photocured under argon atmosphere more effectively suppress mechanical vibrations, most likely due to higher cross-linking density and their overall viscoelastic properties. Analyzing the influence of catalyst concentration on the damping properties of polymer networks, that the highest values of tan *δ* (at the reference temperature point of 37 °C) were noted for samples where 0.5 mol% catalyst was used during the macromonomer synthesis. This result is correlated with these samples having the highest values of gel fraction, lowest swelling degrees, and the macromonomers having the highest acrylation degrees.

### 3.8. Cell viability

**Fig. 13:**
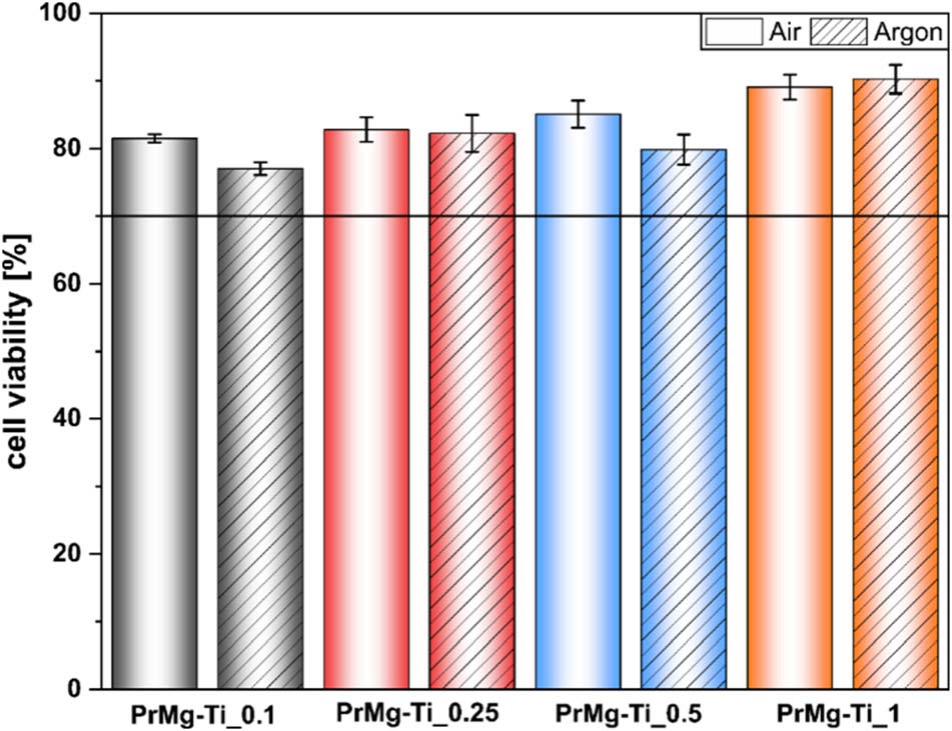
Cell viability L929 cell viability after 24 hours of exposure to extracts of materials obtained with use of various concentrations of Mg–Ti butoxide catalysts.

In order to assess the suitability of the obtained elastomeric photopolymerized networks for potential medical applications, we screened photocured films for cytotoxicity using the extract assay according to ISO 10993-5. For all catalyst concentrations the cell viability results were similar (Fig. 13) and all well above 70%, the cytotoxicity threshold set in ISO 10993-5. Importantly, in all cases robust growth was observed and cell morphology was normal (representative phase contrast photomicrographs are available in Fig. S8†). It is also noteworthy that in this case we did not observe any significant effect of the photocuring atmosphere, air *vs.* argon. This indicates that the effect of oxygen inhibition was relatively modest and is consistent with the gel fraction results which were similar for both photocuring atmospheres. From an application standpoint, this is particularly important, because *in situ* photocuring is much easier to perform under room air, rather than specialized inert gas atmosphere. Overall, these results are similar to those in our previous work with non-toxic homometallic Bi and Zn catalyzed macromonomers and in contrast to the cytotoxic response observed for toxic DBTDL organotin catalyst.^17^ Interestingly, this is despite significantly lower gel fraction for the materials tested here, which suggests that in the previous work indeed the toxicity of residual DBTDL catalyst is likely the main driving factor of cytotoxic response, rather than residual macromonomers.

## 4 Conclusions

The heterometallic Mg–Ti butoxide catalyst was successfully used to synthesize telechelic macromonomers containing urethane-ester cleavable linkages and methacrylic photoreactive terminal groups suitable for photopolymerization towards elastomeric polymer networks. The concentration of the catalyst during the synthesis of macromonomers strongly influenced the reaction kinetics, degree of acrylation, average molecular weight, and dynamic viscosity of the obtained products. As a result, we observed marked differences in the physico-chemical, thermal, and thermomechanical properties of elastomeric films obtained during photopolymerization of the macromonomers. The curing atmosphere (air *vs.* argon) also had effect, particularly on the dynamic thermomechanical properties of obtained polymer networks. Importantly, all of the obtained materials demonstrated high cell viability in extract tests according to ISO 10993-5, independent of the catalyst concentration and curing atmosphere. As a result, we conclude that the heterometallic Mg–Ti butoxide catalyst can be a good candidate for replacement of homometallic catalysts for the synthesis of urethane-based precursors for photocured elastomeric networks for soft tissue biomedical applications.

## Author contributions

Malwina J. Niedźwiedź – investigation, data curation, visualization, writing – original draft, Wojciech Ignaczak – methodology, writing – review & editing, Peter Sobolewski – methodology, investigation, writing – review & editing, Agata Goszczyńska – writing – review & editing, Gokhan Demirci – conceptualization, methodology, writing – review & editing, Miroslawa El Fray – conceptualization, supervision, writing – review & editing, funding acquisition.

## Conflicts of interest

M. El Fray is co-inventor of patents that are licensed to PolTiss Sp. z o.o. G. Demirci is Chief Technology Officer of PolTiss Sp. z o.o. which is commercializing photocrosslinkable biomaterials.

## Supplementary Material

RA-013-D3RA02157B-s001

RA-013-D3RA02157B-s002
