# Supplementary material for: Injectable and photocurable macromonomers synthesized using a heterometallic magnesium–titanium metal–organic catalyst for elastomeric polymer networks
Source: RSC Adv. 2023 Jun 19;13(27):18371–81. doi: 10.1039/d3ra02157b (PMC10277904; doi:10.1039/d3ra02157b)
Supplement: RA-013-D3RA02157B-s002 [file RA-013-D3RA02157B-s002.pdf]

Electronic Supplementary Material (ESI) for RSC Advances.
This journal is © The Royal Society of Chemistry 2023

Injectable and photocurable macromonomers synthesized using a heterometallic magnesium-titanium metal-organic catalyst for elastomeric polymer networks

*Malwina J. Niedźwiedź, Wojciech Ignaczak, Peter Sobolewski, Agata Goszczyńska, Gokhan Demirci**, Miroslawa El Fray**

Department of Polymer and Biomaterials Science, Faculty of Chemical Technology and Engineering, West Pomeranian University of Technology in Szczecin, Al. Piastow 45, 70-311 Szczecin, POLAND

*Corresponding author:: mirfray@zut.edu.pl

**Co-corresponding author: gdemirci@zut.edu.pl

SUPPORTING INFORMATION

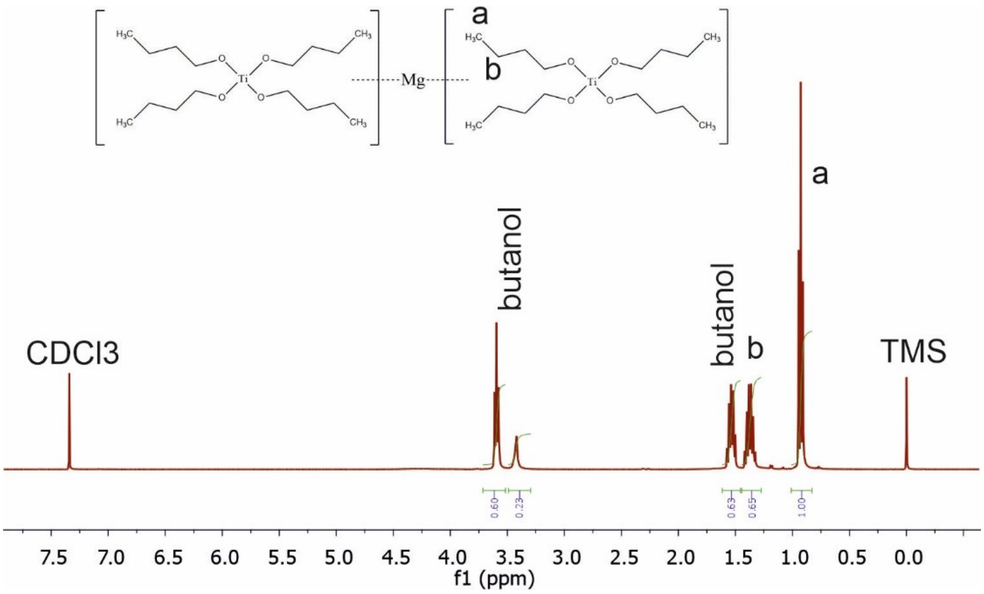

Figure S1 ^{1}H NMR of Mg-Ti butoxide catalyst

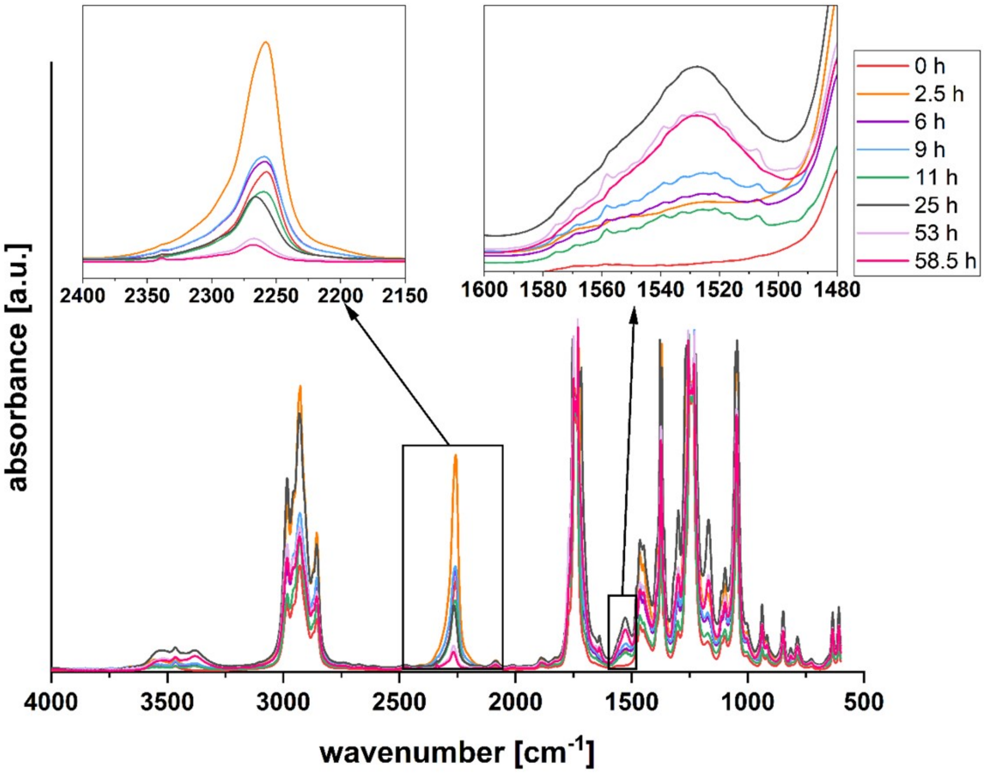

Figure S2 Progress of the reaction with use of 0.1 mol% of catalyst

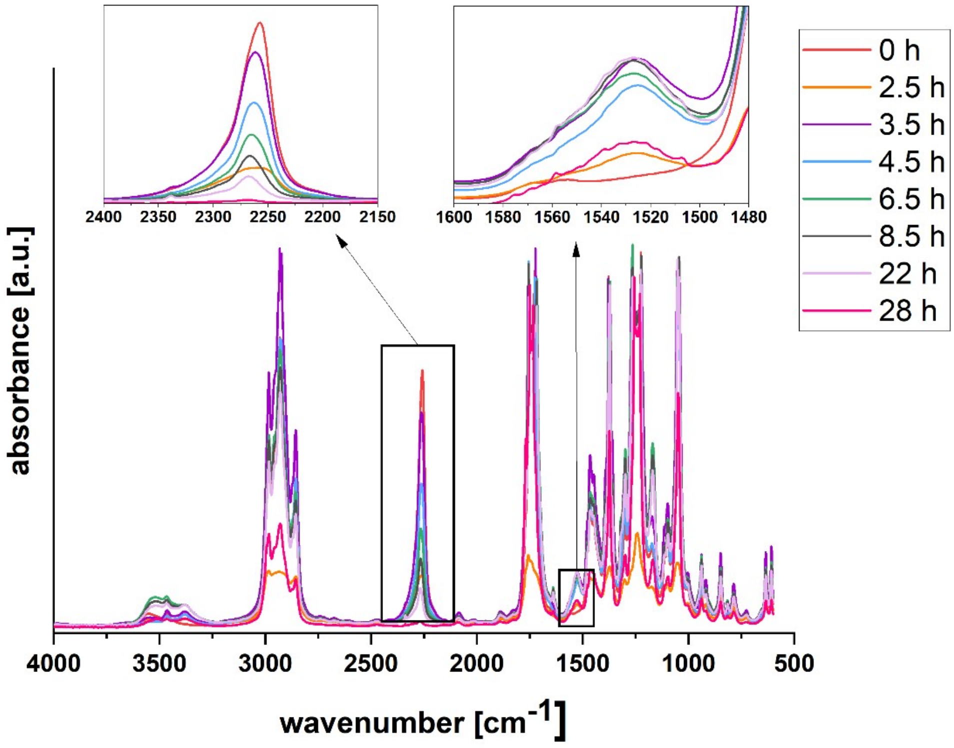

Figure S3 Progress of the reaction with use of 0.25 mol% of catalyst

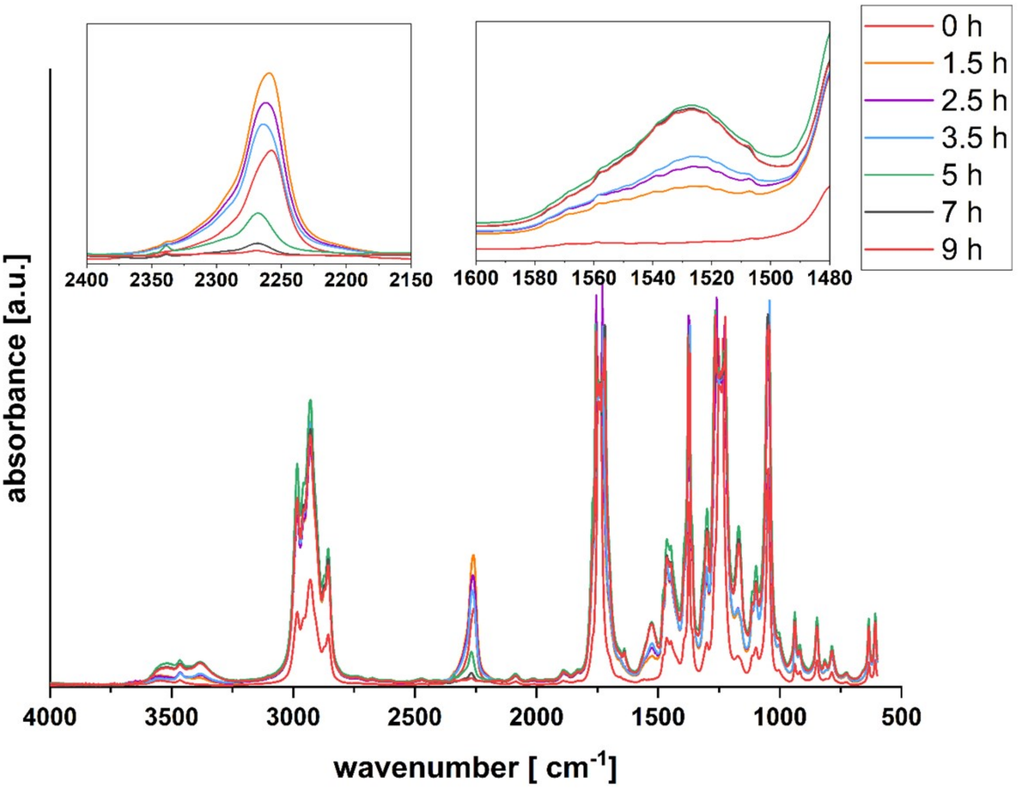

Figure S4 Progress of the reaction with use of 0.5 mol% of catalyst

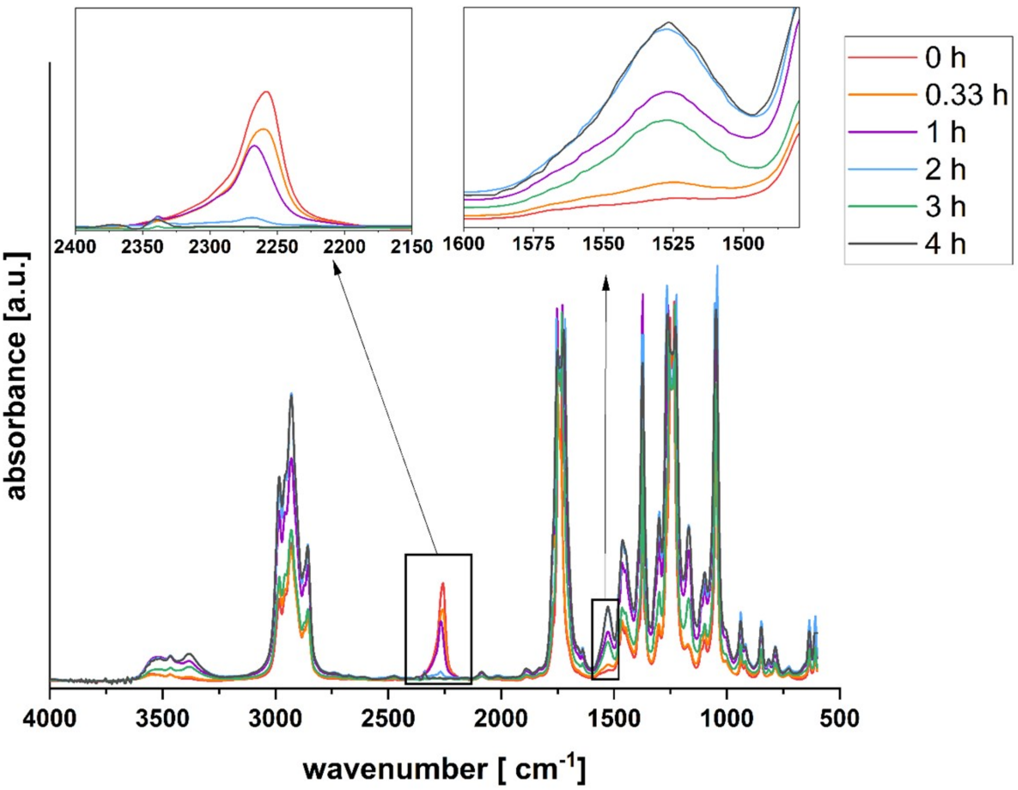

Figure S5 Progress of the reaction with use of 1 mol% of catalyst

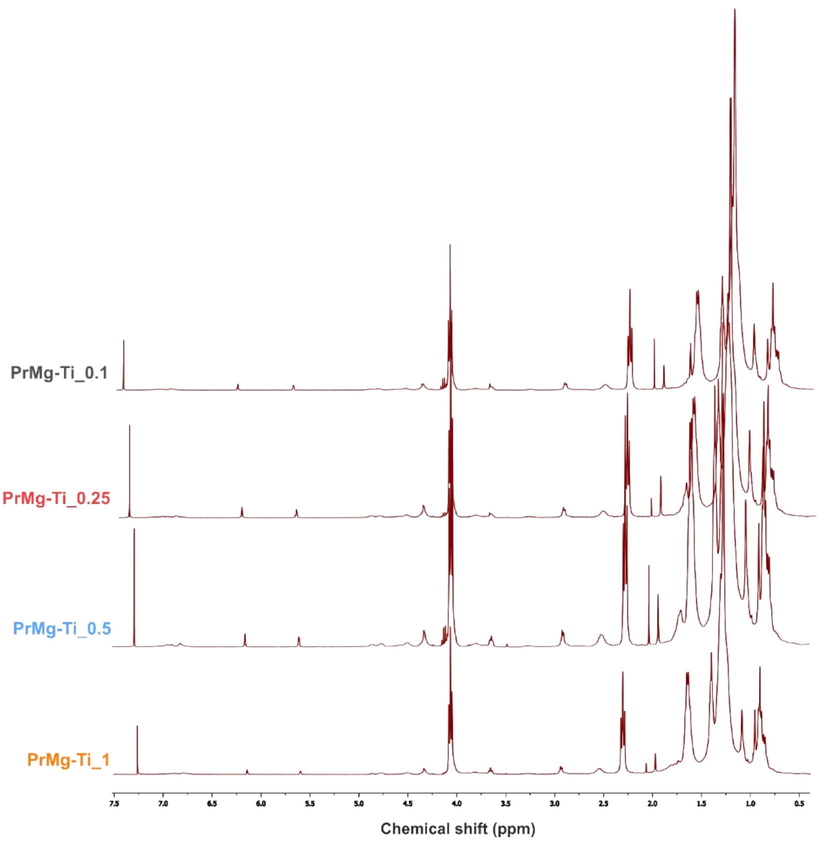

Figure S6 ^{1}H NMR of all macromonomers obtained with use of different catalyst concentrations.

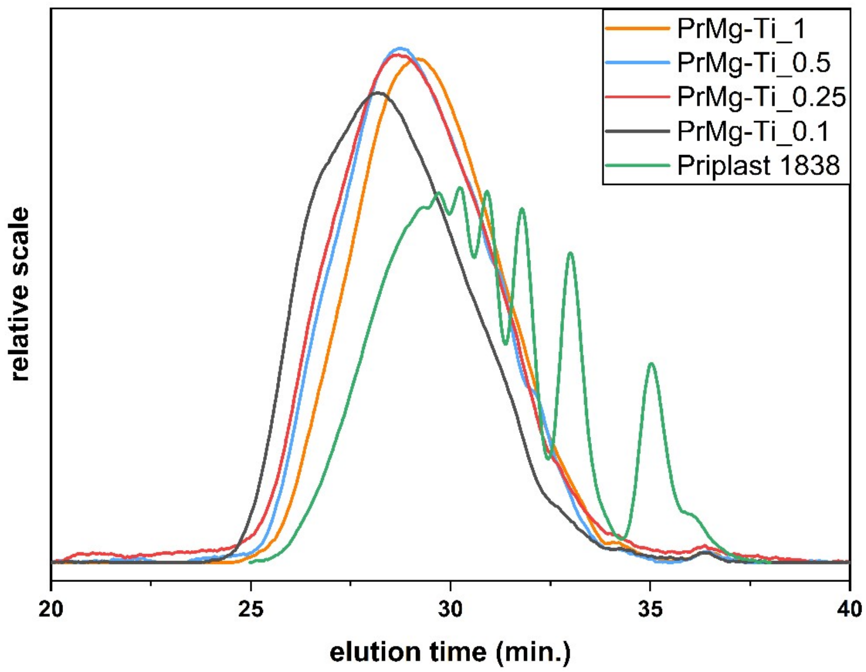

Figure S7 GPC traces of the materials obtained with use of different catalyst concentrations.

Table S1 Degree of acrylation

Catalyst concentration (mol%)	δu,w (1H)	δa (4H)	Degree of Acrylation
0.10	3.40	100	0.136
0.25	3.10	100	0.124
0.50	4.89	100	0.196
0.75	2.54	100	0.102
1.00	2.04	100	0.082

Table S2 DSC results

Material	T_g [°C]	ΔC_p [J/g·°C]
**PrMg-Ti_0.1**	-51.6	0.331
**PrMg-Ti_0.25**	-50.3	0.360
**PrMg-Ti_0.5**	-51.9	0.399
**PrMg-Ti_1**	-51.0	0.346

Table S3 Gel fraction (G_f) of photocured polymer networks in air and in argon

Material	Gel fraction [%]	
	Cured in air	Cured in argon
PrMg-Ti_0.1	65±3	66±4
PrMg-Ti _0.25	57±2	60±1
PrMg-Ti_0.5	75±1	76±2
PrMg-Ti_1	45±2	48±2

Table S4 Swelling test results of obtained thin films

Material	Swelling [%]	
	Cured in air	Cured in argon
PrMg-Ti_0.1	435±23	353±3
PrMg-Ti_0.25	503±10	450±5
PrMg-Ti_0.5	382±8	308±3
PrMg-T _1	488±7	434±10

Table S5 DMTA results

Material	Curing atmosphere	T_g (E''$_{max}$) [°C]	T_g (tan δ_{max}) [°C]	E' at 37 °C [MPa]	E'' at 37°C [MPa]	Tan δ at 37 °C [MPa]
PrMg-Ti _0.1	Air	-36.9	-28.1	0.078	0.014	0.14
	Argon	-35.6	-26.0	0.200	0.067	0.330
PrMg-Ti _0.25	Air	-33.2	-21.8	0.064	0.021	0.21
	Argon	-36.6	-28.3	0.450	0.134	0.296
PrMg-Ti _0.5	Air	-38.9	-30.4	0.045	0.022	0.022
	Argon	-39.7	-31.5	0.058	0.031	0.525
PrMg-Ti _1	Air	-38.5	-28.3	0.049	0.014	0.14
	argon	-38.6	-29.5	0.017	-	-

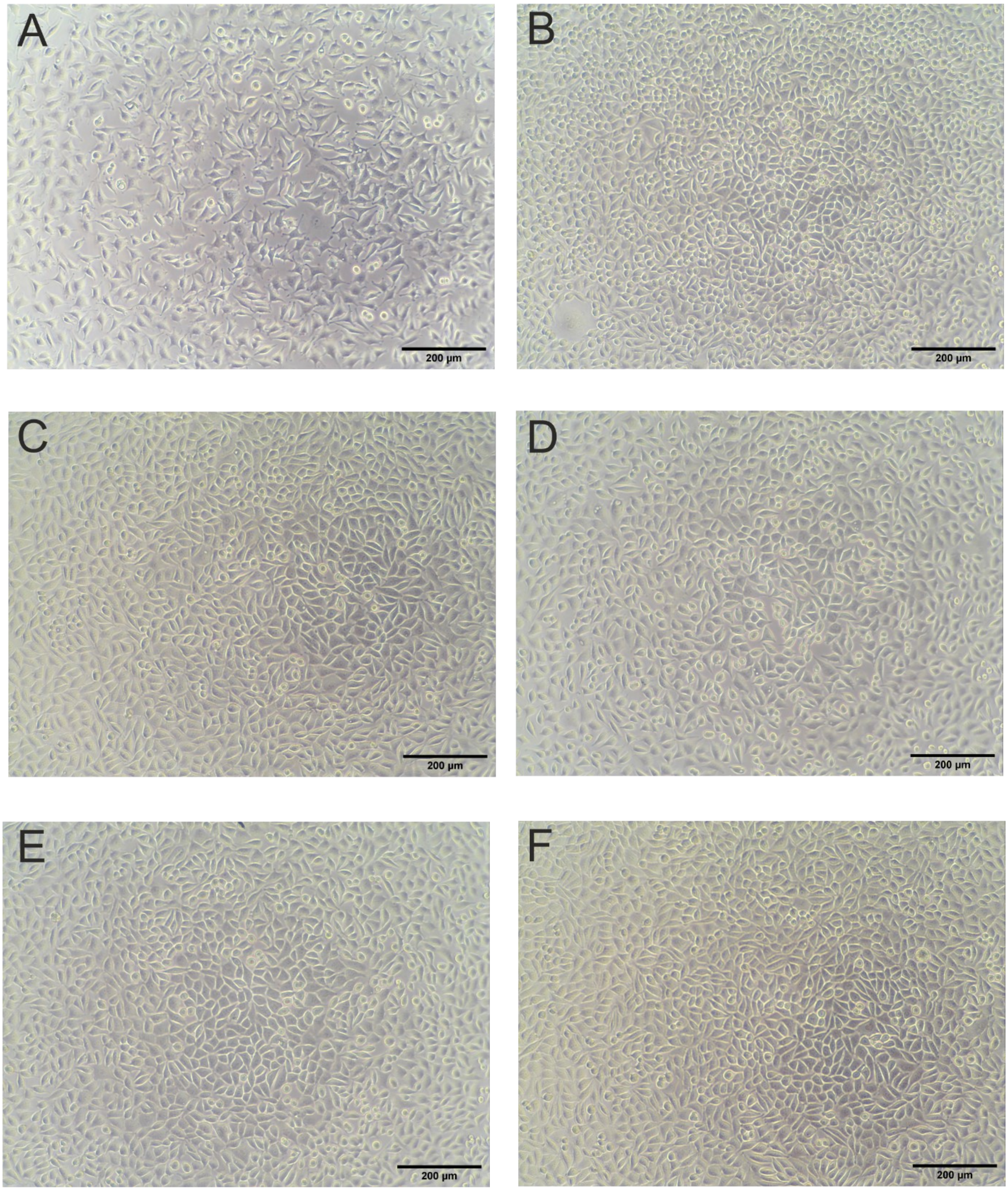

Figure S8 Representative photomicrographs of L929 cells. A) 24 hours after seeding; B) after 24 hours of culture with sham extract; C) after 24 hours of culture with extract of PrMg-Ti_0.1; D) after 24 hours of culture with extract of PrMg-Ti_0.25; E) after 24 hours of culture with PrMg-Ti_0.5; F) after 24 hours of culture with extract of PrMg-Ti_1. All materials were crosslinked in air atmosphere. Scale bars represent 200 µm.
